# Prosopagnosia, Other Specific Cognitive Deficits, and Behavioral Symptoms: Comparison between Right Temporal and Behavioral Variant of Frontotemporal Dementia

**DOI:** 10.3390/vision6040075

**Published:** 2022-12-13

**Authors:** Christos Koros, Ion Beratis, Stavroula Matsi, Anastasia Bougea, Anastasios Bonakis, Ioannis Papatriantafyllou, Efthalia Angelopoulou, Elisabeth Kapaki, Leonidas Stefanis, Sokratis G. Papageorgiou

**Affiliations:** 1 1st Department of Neurology, Eginition Hospital, Medical School, National and Kapodistrian University of Athens, 11528 Athens, Greece; chkoros@gmail.com (C.K.); ioanas96@hotmail.com (I.B.); stmatsi@hotmail.com (S.M.); annita139@yahoo.gr (A.B.); jpapatriantafyllou@gmail.com (I.P.); ekapaki@med.uoa.gr (E.K.); lstefanis@med.uoa.gr (L.S.); sokpapa@med.uoa.gr (S.G.P.); 2 Deree-The American College of Greece, 15342 Athens, Greece; 3 2nd Department of Neurology, Attikon Hospital, Medical School, National and Kapodistrian University of Athens, 12462 Athens, Greece; bonakistasos@med.uoa.gr

**Keywords:** frontotemporal dementia, right temporal atrophy, frontal atrophy, prosopagnosia, behavioral symptoms

## Abstract

Right temporal variant of frontotemporal dementia (rtv-FTD) represents an uncommon and recently described frontotemporal dementia (FTD) entity presenting with symptoms in many ways comparable to those of the frontal or behavioral variant of FTD (bv-FTD). The aims of this study were to explore the timing of cognitive and behavioral symptoms of rtv-FTD, and to compare the distinct cognitive deficits including prosopagnosia and behavioral symptoms of rtv-FTD patients with those observed in bv-FTD patients. We reviewed the records of 105 patients clinically diagnosed with FTD. A total of 7 patients (5 men/2 women) with FTD and marked right temporal atrophy in magnetic resonance imaging (MRI) were detected. Clinical features were compared with those observed in a group of 22 age-matched patients (16 men/6 women) with FTD and predominant frontal lobe atrophy. The main presenting symptoms of rtv-FTD were prosopagnosia, apathy, and episodic memory impairment. In contrast, social awkwardness and compulsive behaviors were dominant in later stages of the disease together with disinhibition and loss of insight with a marked personality change. Although the cognitive and behavioral profiles of patients with right temporal or frontal lobes atrophy present substantial similarities, each subtype has a number of distinct characteristics. It appears that prosopagnosia, obsessive behaviors, and psychotic symptoms are more prominent in rtv-FTD patients.

## 1. Introduction

Frontotemporal dementia (FTD) or frontotemporal lobar degeneration (FTLD) represents the second most common cause of degenerative dementias with presenile onset. The pathological substrate of these disorders is heterogeneous and various molecular mechanisms (Tau or TARDNA-Binding Protein 43 (TDP43) pathology among others) have been implicated in the pathogenesis of the disease [1]. Miscellaneous clinical phenotypes have been described in FTD patients including apathetic symptoms, disinhibition, emotional disturbance, speech disorders, cognitive impairment, and socially deviating behavior [1]. Three major subtypes of the disorder have been characterized so far: the frontal or behavioral variant of FTD (bv-FTD) exhibiting mainly atrophy of the frontal lobes, the semantic variant (sv-FTD) with predominant left temporal lobe atrophy, and nonfluent progressive aphasia (PnFA) with left perisylvian atrophy. Each subtype has been associated with certain distinct cognitive or behavioral deficits [2,3].

Although left-sided atrophy syndromes have been described adequately, literature evidence concerning the clinical spectrum of FTD patients with predominantly right-sided atrophy are limited. Selective left temporal lobe atrophy results in semantic aphasic disorders (knowledge for words and objects) with a subtle initial involvement of social skills and behavior. In contrast, the right temporal variant of FTD (rtv-FTLD) represents an emerging subgroup of FTD, characterized by a selective or predominant atrophy of the right temporal lobe. The hallmarks of this subtype include impaired semantic knowledge for faces (prosopagnosia), as well as behavioral deficits such as emotional blunting, loss of empathy, and socially inert behavior [4,5]. Nevertheless, it is uncertain if the lobar atrophy itself or rather its impaired connectivity with other brain areas is responsible for the clinical manifestations of the disorder. Magnetic resonance imaging (MRI) and single-photon emission computerized tomography (SPECT) studies underscored a selective or predominant atrophy of the right temporal lobe, although left temporal and frontal lobe atrophy can coexist to a lesser extent [4,5,6]. The main affected areas include a circuit comprising the right mesial temporal cortex, the parahippocampal gyrus, the right fusiform gyrus, and the orbitofrontal cortex.

Previous reports concerning rtv-FTD involve case reports that described the pivotal symptoms of this variant and paved the way for its characterization as a relatively novel entity [5,6,7]. One of the most extensive studies in rtv-FTD was published by Chan et al. [4], who compared the features of 20 right-sided atrophy FTD patients with those of 10 left-sided counterparts and concluded that a core profile of prosopagnosia, spatial disorientation, episodic memory impairment, social awkwardness, and apathy characterized the former. A number of other studies also focused on the differences between left and right temporal atrophy variants showing that speech-related deficits were more common in the former, and behavioral disturbances in the latter [8,9]. In this way, the behavioral pattern of rtv-FTD seems to be close to that seen in bv-FTD.

The aim of the present study was to define the timing of cognitive and behavioral symptoms of the rtv-FTD disorder and to assess the distinct cognitive and behavioral profile of rtv-FTD patients as compared to patients suffering from bv-FTD. To the best of our knowledge, despite the presence of literature comparing the symptomatology of right versus left temporal atrophy, this is one of the first studies focusing on the detailed clinical features of right temporal versus frontal lobe atrophy patients.

## 2. Materials and Methods

**Figure 1:**
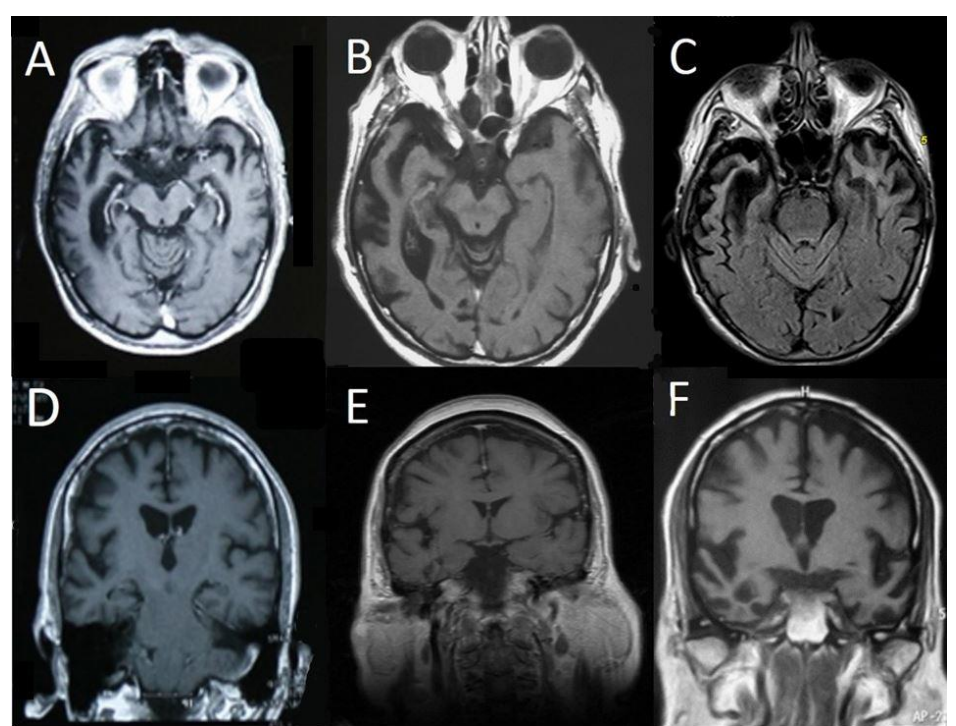
Representative Magnetic Resonance Imaging (MRI) scans of 6 patients from the right temporal variant of frontotemporal dementia (rtv-FTD) group. T1 axial (**A**,**B**) and fluid attenuated inversion recovery (FLAIR) (**C**) scans show severe right anterior temporal lobe atrophy with milder involvement of the left temporal lobe. T1 coronal (**D**,**E**) scans show a less pronounced but predominant right temporal lobe atrophy, whereas scan (**F**) shows bilateral involvement with predominant right temporal lobe atrophy.

We reviewed the records of 105 patients diagnosed with FTD, examined at the Cognitive–Extrapyramidal disorders Unit of the 1st and the 2nd Neurology Department, of the National and Kapodistrian University of Athens in Greece (2009–2016). Diagnosis was made by senior neurologists specialized in cognitive disorders based on the diagnostic criteria, after a comprehensive neurological, neuropsychological, and neuroimaging evaluation. Selection criteria of rtv-FTD patients were patients with a clinical diagnosis of FTD and predominant lateral temporal lobe atrophy in their first available brain MRI. A total of 7 patients (5 men/2 women) with a clinical diagnosis of FTD and predominant lateral right temporal atrophy in MRI were detected in this sample. Clinical features of these patients were compared with those observed in a group of 22 age-matched patients (16 men/6 women) with a clinical diagnosis of FTD and predominant frontal lobe atrophy in their first available brain MRI [2]. Patients were designated as having predominant lateral right temporal or frontal atrophy according to their initial available brain MRI scans which were blindly assessed by experienced neuroradiologists as described in previous studies [8]. In the majority of cases of rtv-FTD, a certain degree of bilateral temporal lobe atrophy was present with a marked predominance of atrophy on the right side (Figure 1).

In the two groups of patients, demographic data (age), age at onset, disease duration, behavioral symptoms, and scores at neuropsychological testing were assessed and compared. The age at onset was set as the age at which patients exhibited the first cognitive or behavioral symptom that was clinically related to FTD. Disease duration for both rtv-FTD and bv-FTD subgroups was defined from the appearance of the first cognitive or behavioral symptom that was clinically related to FTD until our first evaluation. All patients had undergone a comprehensive neurobehavioral evaluation including clinical assessment, interviews with their family members (or caregivers), neuropsychological testing, and behavioral assessment.

Neuropsychological testing included the following scales: Mini Mental State Examination test (MMSE), Modified Mini Mental Status Examination (mMMSE) [10] including 5-words memory test [11] and 5-objects memory test [12], and Frontal Assessment Battery (FAB) [13]. Behavioral assessment included the following scales and inventories: NeuroPsychiatric Inventory (NPI) [14] and Frontal Behavioral Inventory (FBI) [15]. 

Clinical Dementia Rating (CDR) [16] along with interviews with the patient and his/her caregivers were used for the assessment of the episodic memory impairment and the definition of the dementia stage (including the computation of the sum of boxes score). The Instrumental Activities of Daily Living (IADL) was used for the assessment of the functional impairment of the patients [17].

The presence of symptoms was defined by using both clinical data (interviews with caregivers) and neurobehavioral scales (NPI, FBI scales). Described symptoms were classified according to their nature (cognitive or behavioral) and timing as early onset (<1 year since disease appearance) or late onset. Behavioral symptoms were further categorized in three major subgroups: (a) symptoms in the spectrum of apathy (loss of empathy, loss of initiative, loss of interests), (b) symptoms in the spectrum of loss of self-control (disinhibition, inappropriate laughter, irritability, aggressiveness, impulsivity), and (c) symptoms in the spectrum of behavioral rigidity symptoms (obsessive-compulsive behavior). Other symptoms concerning psychiatric disorders (psychosis, depression, euphoria), food-related disorders, personal hygiene, and sleep were assessed separately.

### Statistical Analysis

Categorical parameters were analyzed using chi-square test for each cognitive or behavioral symptom separately, and continuous data using one way ANOVA with the FTD subtype as the independent variable. Statistical significance was set at *p* value < 0.05.

## 3. Results

### 3.1. Prevalence and Timing of Symptoms in the rtv-FTD Group

In the rtv-FTD group, the mean age was 72.5 ± 5.4, the mean onset age was 70 ± 6.5 years (range: 62 to 82 years), and the mean disease duration (from the appearance of the first cognitive or behavioral symptom that was clinically related to FTD until our first evaluation) was 2.5 ± 0.9 years. Mean MMSE score at presentation was 21.2 ± 8.2. Initial cognitive symptoms included prosopagnosia (5/7), mild episodic memory impairment (4/7), naming and word finding problems (1/7), and notably voice recognition deficits (phonagnosia) (1/7) (Table 1).

As the disease progressed, practically all patients experienced memory deficits and prosopagnosia. Executive dysfunction was then present in 6/7 patients and speech related problems also increased (3/7). Disorientation in space was demonstrated in 2/7 patients.

Behavioral symptoms were largely heterogeneous during the first year of the disorder, including emotional blunting (4/7), depression (1/7), irritability (2/7), and unprovoked laughter (1/7). Apathy appeared to be the prevailing early behavioral symptom both in terms of emotional (indifference) and motivational apathy (lack of initiative) (5/7).

The most marked late onset behavioral symptoms were disinhibition and loss of insight, both present in most patients (5/7). Aggressiveness or sexually deviant behaviors were also described. Sexually deviant behavior included hypersexuality and rude comments or gestures with sexual content (2/7). Most patients exhibited irritability (5/7), while visual hallucinations (unfamiliar people invading their house) (2/7), altered food preference with consumption of increased amount of food and sweet craving (4/7), deterioration of personal hygiene (3/7), and changes in sleep schedule (insomnia) (2/7) were also noted. Obsessive and compulsive behaviors were common, observed in 5/7 patients, including obsession with number-based games (sudoku) and hyper religiosity.

### 3.2. Comparison between rtv-FTD and bv-FTD Patients

Detailed demographic and neuropsychological data of rtv-FTD and bv-FTD patients are provided in Table 2.

Age, age at onset, and disease duration did not significantly differ between rtv-FTD and bv-FTD subgroups. The mean MMSE and mMMSE scores did not differ. On the other hand, bv-FTD patients exhibited a lower FAB score, a finding in accordance with the prominent frontal lobe involvement in this group. No marked difference could be observed as far as CDR, IADL, or NPI scores were concerned.

The prevalence of the majority of cognitive symptoms (executive functions and speech) was similar in rtv-FTD and bv-FTD patients (Table 3).

Disorientation in time and space was observed in almost 30% of patients in both groups. Episodic memory was impaired in 86% of late stage rtv-FTD vs. 59% of their frontal counterparts, but the difference did not reach statistical significance. Speech related disorders occurred to the same extent in both groups as well. Regarding executive functions, attention was attenuated in both groups. Nevertheless, the most prominent cognitive symptom in rtv-FTD was prosopagnosia and this was rarely encountered in frontal lobe atrophy patients (*p* < 0.001).

As far as behavioral symptoms are concerned (Table 4), symptoms in the spectrum of apathy (loss of initiative, loss of interests) were more frequent in rtv-FTD patients than in the frontal lobe group (*p* = 0.016 and 0.008 respectively).

There was not such a difference in apathy. Symptoms related to loss of self-control (disinhibition, inappropriate laughter, aggressiveness, irritability) were of equal predominance in both groups. Behavioral rigidity symptoms were more frequent in rtv-FTD patients, and this difference was significant for obsessive-compulsive behavior (*p* = 0.008).

Food-related disorders were equally recorded in the bv- and rtv-FTD groups. Mood disorders such as depression and euphoria were evenly encountered. Sleep disorders and personal hygiene deterioration appeared to be more frequent in the rtv-FTD subgroup but the difference was not statistically significant. Interestingly, psychotic symptoms (hallucinations and delusions such as the feeling that strangers had invaded their house or the conception that their spouse committed adultery) were noted mainly in rtv-FTD patients and the difference was significant for delusions (*p* = 0.030).

## 4. Discussion

Right temporal variant frontotemporal dementia represents a relatively novel subtype of the disorder probably accounting for roughly less than 10% of the overall cases of FTD according to recent reports [18]. In our cohort, we identified 7 such cases out of a total of 105, i.e., 6.7%. It is possible that it is clinically underdiagnosed due to the often-subtle symptoms encountered during the early stages of the disorder. In contrast, the usually disabling early involvement of speech in semantic dementia with marked left temporal lobe atrophy renders this subtype easier to recognize. A number of previous reports emphasize the distinct cognitive and behavioral profiles of patients with right- or left-sided temporal lobe atrophy [4,9].

In the present study, we attempted to investigate the timing of symptoms in rtv-FTD patients. The main presenting cognitive symptoms of rtv-FTD appeared to be prosopagnosia (5 out of 7 patients) and episodic memory impairment, while the main early behavioral symptom was apathy. Prosopagnosia involved both famous and familiar persons. Difficulty in famous or familiar people identification has been reported previously as a core symptom of rtv-FTD [4,5,6,19,20]. Prosopagnosia has been correlated with atrophy of the inferior pole of the right temporal lobe [19,20]. Caregivers tend to underestimate this symptom and usually consider it to be a part of the overall cognitive decline of the patient. Two of our patients also exhibited difficulty in voice recognition, a rare finding that has also been described previously [21].

Social awkwardness and compulsive behaviors were dominant in later stages of the disease, possibly as a result of disinhibition and loss of insight of patients along with a marked personality change. Behavioral disorders are considered to be a key point of rtv-FTD as shown also on previous reports [4,8]. The right hemisphere is considered to be dominant in the comprehension and expression of emotions [22]. Patients with rtv-FTD exhibited emotional blunting and loss of insight and were often unaware of their emotions and actions. Other studies have also shown that individuals with right temporal lobe dysfunction often show irritability, aggressiveness, and violent or sexually deviant behavior with a great impact on their social or professional life [23]. Finally, we should highlight the possibility of motor neuron disease symptoms in rtv-FTD patients, as was the case in one of our patients after clinical and neurophysiological assessment. Previous reports adequately address this issue [24,25].

The comparison between the symptoms of rtv-FTD and bv-FTD in the present study shed light on important similarities and differences of the two variants. As far as cognitive symptoms are concerned, it seems that most of them are not prevailing characteristics of either rtv-FTD or bv-FTD patients. However, episodic memory deficit was frequently encountered in the initial stages of our rtv-FTD group. A recent report also highlights a certain degree of episodic memory impairment in rtv-FTD, although milder compared to Alzheimer’s disease (AD) patients [26]. In our study, spatial orientation was only moderately impaired in most patients during early disease course. In contrast, in the study of Chan and co-authors, getting lost was encountered in 65% of the rtv-FTD subgroup (13 out of 20 patients) [4]. Finally, prosopagnosia was the most prominent symptom in our rtv-FTD group (found in 6 out of 7 patients). By contrast, prosopagnosia was essentially absent in bv-FTD patients (seen only in 1 out of 22).

Behavioral profiles of patients with right temporal or frontal lobe atrophy present substantial similarities. Apathy and loss of self-control are key points of both disorders and such clinical features render the accurate clinical evaluation challenging [27]. Nevertheless, each subtype has a number of distinct characteristics that might facilitate differential diagnosis. Social awkwardness resulting from loss of empathy was observed to the same extent in rtv-FTD and bv-FTD patients in our study. Atrophy in the right nucleus accumbens, orbitofrontal cortex right superior temporal sulcus, and right mediotemporal limbic structures has been correlated to the development of disinhibition [27,28]. Symptoms in the spectrum of loss of self-control such as irritability, aggressiveness, and difficulty in social adaptation were major traits of both FTD variants studied, and our data corroborate previous studies suggesting that these symptoms cannot be helpful in discriminating the two disorders [4,29,30].

The development of apathy and emotional blunting is an important and early feature of both rtv-FTD and bv-FTD groups. It has been correlated with right caudate nucleus, right middle temporal lobe, and anterior insular region atrophy [31,32]. According to Yassuda et al., atrophy in the right dorsolateral prefrontal cortex has also been implicated in apathy [33].

Obsessive compulsive behavior has been reported in both subgroups but based on our data, it is more pronounced in right temporal lobe atrophy. This is in agreement with literature data reporting that obsessive-compulsive behavior is a hallmark of patients with prominent right temporal lobe atrophy [4,8,34]. This symptom becomes more obvious during the progression of the disease. The disorder might be expressed as impulsiveness, mental rigidity with fixed ideas, and usually compulsive actions leading to a bizarre behavior. Although dysfunction of the frontal lobes appears to play a role in obsessive compulsive behavior, temporal involvement seems to be more crucial [35]. It has been proposed that the attention of rtv-FTD patients is attracted towards verbal or symbolic stimuli (playing cards, word and number-based games, keeping diaries) as opposed to semantic dementia patients with left temporal lobe atrophy, who develop an interest in visual stimuli (bright colors, collecting objects) according to their perceptive function that remains relatively well preserved [8].

Considering mood disorders, we found a similar prevalence of depression or euphoria in both FTD variants assessed [36]. However, psychotic symptoms (illusions and hallucinations) were much more frequent in our rtv-FTD patients. It is notable that psychotic symptoms are reported to be rare in bv-FTD patients [36,37]. Visual hallucinations have also been reported as isolated features of certain rtv-FTD patients (10%, 2 out of 20 patients) in the extensive study by Chan et al. [4]. Binge eating and altered food preferences, such as developing a sweet tooth, have been linked to right orbitofrontal-insular-striatal atrophy in recent reports [38,39]. Similarly, we attested the presence of food-related disorders in half of our rtv-FTD patients, but also in a significant proportion of their bv-FTD counterparts. Other daily habit alterations involving sleep schedule and personal hygiene were equally impaired in both FTD variants.

Our results are in accordance with the recent study of Kamminga and co-authors assessing right lateralized FTD and bv-FTD patients, who suggested that prosopagnosia and obsessive compulsive behaviors prevail in rtv-FTD, whereas emotional disorders, disinhibition, decreased empathy, and diet changes are common in both dementia subtypes [40]. The above findings suggest that rtv-FTD represents a distinct subgroup of FTD that while infrequent, merits recognition as a separate variant in the FTD spectrum. In spite of many former comparative studies between right and left temporal lobe atrophy patients, it appears that differential diagnosis is really challenging when we assess individuals with right temporal lobe atrophy and bv-FTD patients. This stems from substantial similarities and many confounding factors between the two variants [41].

This work has limitations. Lateral temporal and frontal lobe atrophy in MRI scans was assessed visually, and no exact medial temporal lobe atrophy (MTA) score was available to mention and compare between our two subgroups. Some patients had undergone brain MRI of 1,5T and some of 3T. However, neuroimaging evaluation was made by experienced neuroradiologists of the two academic centers, which limits the possibility of wrong assessments. Comparisons of MTA scores between bv-FTD and rtv-FTD would be interesting to explore in future relevant studies. Furthermore, although a thorough neurological examination was made in each patient at least during the initial evaluation, we did not compare specific neurological deficits other than behavioral or cognitive symptoms (such as parkinsonism, primitive reflexes, dysphagia, etc.) between our FTD subgroups. The investigation of such differences would be useful in future studies. Unfortunately, we did not have the opportunity to screen for genetic causes of FTD in our sample, including *TDP43*, *MAPT*, or *C9ORF72*. Nevertheless, future genetic-phenotypic associations and comparisons between bv-FTD and rtv-FTD subgroups would be of particular value. Another limitation is the retrospective nature of this study, which might possibly contribute to recall bias. Moreover, owing to the rarity of the disorder, the number of patients in the rtv-FTD group is small, as was the case in most previous reports. We believe that our study will add in the existing literature, and further pave the way for a more detailed comparative evaluation between FTD patients with frontal and right temporal atrophy.

## 5. Conclusions

Although the cognitive and behavioral profiles of patients with right temporal or frontal lobes atrophy present substantial similarities, each subtype has a number of distinct characteristics. It seems that prosopagnosia, obsessive behaviors, and psychotic symptoms are more prominent in rtv-FTD patients. Future studies investigating other neurological signs and specific genetic forms of FTD would enhance our understanding of the clinical and pathophysiological differences of these specific FTD subtypes.

## Figures and Tables

**Table 1:** Main early-onset and late-onset cognitive and behavioral symptoms of right temporal variant of frontotemporal dementia (rtv-FTD) patients, with the cut-off of one year since the appearance of the first cognitive or behavioral symptom clinically related to frontotemporal dementia (FTD).

Early Onset	Late Onset
Prosopagnosia 71% (5) *	Prosopagnosia 86% (6)
**Memory impairment 57% (4)**	Memory impairment 86% (6)
**Logopenic speech 14% (1)**	Attention impairment 71% (5)
**Voice recognition 14% (1)**	Logopenic speech 43% (3)
**Apathy 71% (5)**	Apathy 100 % (7)
**Emotional blunting 57% (4)**	Emotional blunting 86% (6)
**Irritability 29% (2)**	Loss of interest 71% (5)
**Inappropriate laughter 14% (1)**	Disinhibition 71% (5)
**Depression 14% (1)**	Delusions 71% (5)
	Obsessions 71% (5)
	Compulsive behavior 57% (4)
	Aggressiveness 57% (4)
	Behavioral rigidity 57% (4)
	Dietary changes 57% (4)

* The percentage describes the rate of appearance of each symptom in the group of rtv-FTD patients. In the parenthesis, the number of patients experiencing each symptom is mentioned.

**Table 2:** Demographic and neuropsychological data in right temporal variant of frontotemporal dementia (rtv-FTD) and behavioral variant of frontotemporal dementia (bv-FTD) patients in our initial evaluation.

	rtv-FTD (N = 7)	bv-FTD (N = 26)	Statistical Significance
**Age**	72.5 ± 5.4	69.2 ± 6.7	ns
**Age at onset**	70 ± 6.5	66.4 ±7.2	ns
**Disease duration**	2.5 ± 0.9	3.2 ± 1.2	ns
**MMSE**	21.2 ± 8.2/30	16.3 ± 6.7/30	ns
**mMMSE**	32.5 ± 13.4/57	30.4 ± 11.8 /57	ns
**FAB**	11 ± 2.6/18	7 ± 3.1 /18	*p* < 0.05
**NPI**	17 ± 7.5	18 ± 16	ns
**CDR**	8 ± 5.6	7 ± 5	ns
**IADL**	10 ± 6.9	11 ± 6.7	ns
**FBI**	29 ± 15.3	20 ± 12	ns

Data were given in (Means ± SD), MMSE: Mini Mental State Examination test; mMMSE: modified Mini Mental Status Examination; FAB: Frontal Assessment Battery; NPI: NeuroPsychiatric Inventory; CDR: Clinical Dementia Rating; IADL: Instrumental Activities of Daily Living; FBI: Frontal Behavioral Inventory

**Table 3:** Cognitive symptoms in right temporal variant of frontotemporal dementia (rtv-FTD) versus behavioral variant of frontotemporal dementia (bv-FTD) patients in our initial evaluation.

	rtv-FTD	bv-FTD	Statistical Significance
**Episodic memory impairment**	86 % (6) *	59% (13)	ns
**Attention impairment**	71 % (5)	36 % (8)	ns
**Concentration impairment**	14 % (1)	5% (1)	ns
**Disorientation in space**	29 % (2)	30 % (8)	ns
**Aphasia**	29 % (2)	55 % (12)	ns
**Logopenic speech**	43 % (3)	55 % (12)	ns
**Prosopagnosia**	86 % (6)	5% (1)	*p* < 0.001

* The percentage describes the rate of appearance of each symptom within each group. In the parenthesis, the number of patients within each group experiencing each symptom is mentioned.

**Table 4:** Behavioral symptoms in right temporal variant FTD versus behavioral variant FTD patients in our initial evaluation.

	rtv-FTD	bv-FTD	Statistical Significance
**Apathy**	100 % (7) *	64 % (14)	ns
**Loss of interest**	71 % (5)	14 % (3)	*p* < 0.01
**Loss of initiative**	71 % (5)	18 % (4)	*p* < 0.05
**Disinhibition**	71% (5)	59% (13)	ns
**Irritability**	86% (6)	50% (11)	ns
**Aggressiveness**	57% (4)	40% (9)	ns
**Inappropriate laughter**	29% (2)	18% (4)	ns
**Behavioral rigidity**	57% (4)	27% (6)	ns
**Obsessions**	71% (5)	14% (3)	*p* < 0.05
**Compulsive behavior**	57% (4)	9% (2)	*p* < 0.05
**Depression**	14% (1)	32% (7)	ns
**Euphoria**	29% (2)	23% (5)	ns
**Delusions**	71% (5)	14% (3)	*p* < 0.05
**Hallucinations**	29% (2)	4% (1)	ns
**Dietary changes**	57% (4)	36% (8)	ns
**Sleep problems**	29% (2)	9% (2)	ns
**Personal hygiene**	43% (3)	35% (9)	ns

* The percentage describes the rate of appearance of each symptom within each group. In the parenthesis, the number of patients within each group experiencing each symptom is mentioned.

## Data Availability

The data presented in this study are available in the article.
